# Clinical evaluation, biochemistry and genetic polymorphism analysis for the diagnosis of lactose intolerance in a population from northeastern Brazil

**DOI:** 10.6061/clinics/2016(02)06

**Published:** 2016-02

**Authors:** Paulo Roberto Lins Ponte, Pedro Henrique Quintela Soares de Medeiros, Alexandre Havt, Joselany Afio Caetano, David A C Cid, Mara de Moura Gondim Prata, Alberto Melo Soares, Richard L Guerrant, Josyf Mychaleckyj, Aldo Ângelo Moreira Lima

**Affiliations:** I Universidade Federal do Ceará, Instituto de Biomedicina do Semi-Árido Brasileiro (IBISAB) & Departamento de Fisiologia e Farmacologia, Fortaleza/, CE, Brazil; II University of Virginia, Center for Global Health and Center for Public Health Genome, Charlottesville, Virginia, US

**Keywords:** Lactose Intolerance, Single Nucleotide Polymorphisms, Clinical Management, Diagnosis of Lactose Intolerance, Lactose-Tolerant Phenotype

## Abstract

**OBJECTIVE:**

This work aimed to evaluate and correlate symptoms, biochemical blood test results and single nucleotide polymorphisms for lactose intolerance diagnosis.

**METHOD:**

A cross-sectional study was conducted in Fortaleza, Ceará, Brazil, with a total of 119 patients, 54 of whom were lactose intolerant. Clinical evaluation and biochemical blood tests were conducted after lactose ingestion and blood samples were collected for genotyping evaluation. In particular, the single nucleotide polymorphisms C>T_-13910_ and G>A_-22018_ were analyzed by restriction fragment length polymorphism/polymerase chain reaction and validated by DNA sequencing.

**RESULTS:**

Lactose-intolerant patients presented with more symptoms of flatulence (81.4%), bloating (68.5%), borborygmus (59.3%) and diarrhea (46.3%) compared with non-lactose-intolerant patients (*p*<0.05). We observed a significant association between the presence of the alleles T_-13910_ and A_-22018_ and the lactose-tolerant phenotype (*p*<0.05). After evaluation of the biochemical blood test results for lactose, we found that the most effective cutoff for glucose levels obtained for lactose malabsorbers was <15 mg/dL, presenting an area under the receiver operating characteristic curve greater than 80.3%, with satisfactory values for sensitivity and specificity.

**CONCLUSIONS:**

These data corroborate the association of these single nucleotide polymorphisms (C>T_-13910_ and G>A_-22018_) with lactose tolerance in this population and suggest clinical management for patients with lactose intolerance that considers single nucleotide polymorphism detection and a change in the biochemical blood test cutoff from <25 mg/dL to <15 mg/dL.

## INTRODUCTION

Lactose intolerance (LI) is a common clinical syndrome experienced by people worldwide. It comprises a variety of symptoms, such as abdominal pain, flatulence, bloating, borborygmus and osmotic diarrhea, caused by the breakdown of nondigested lactose by the gut microflora 1.

Nondigestion of lactose, frequently referred to as lactose malabsorption (LM), is due to low expression of lactase. This condition is also called hypolactasia or lactase nonpersistence and is a physiologic feature occurring in most mammals later in life 2. Lactase persistence varies among different human populations, ranging from 95% in White northern Europeans and North Americans to approximately 50% or less in South America and African countries, such as Cameroon, Mali and South Africa, to nearly 0% in certain Asian countries, including China 3,4.

Interestingly, not all patients with LM will develop symptoms of LI. Therefore, usual LI management involves excluding milk and milk products from the diet, which can lead to an imbalance of calcium in the body 5,6.

LI can be diagnosed through several approaches. The gold standard is the measurement of lactase, sucrose and maltase activity through intestinal biopsies. However, this method is not commonly used due to its invasive nature 2. Other tests, such as the lactose breath test, biochemical blood tests and colorimetric-based reactions of biopsies (or quick tests), are more frequently used 7,8.

The literature reports that in addition to biochemical blood tests, genetic markers may be useful for LI diagnosis. Two major markers have been identified so far: the single nucleotide polymorphisms (SNPs) C>T_-13910_ and G>A_-22018_, located upstream of the lactase gene. Both have been associated with lactase persistence in several populations 4,9. In Brazil, most studies have evaluated southeastern and southern populations 10,11; only one study has investigated northeastern populations, but it lacked a comparative clinical approach 12. Further studies are thus needed to investigate whether these polymorphism associations occur in different regions of Brazil, including populations in the Northeast.

In the present study, we had the following objectives: to evaluate SNPs for LI diagnosis in a Brazilian population from Fortaleza, Ceará, to correlate symptoms with biochemical blood test results and to determine the associations between cutoff values for the biochemical blood test and both symptoms and SNPs.

## MATERIALS AND METHODS

### Location

The study was conducted at the Universidade Federal do Ceará (UFC), Hospital Universitário Walter Cantidio, between January and August 2010.

### Study type and ethical review board approval

A cross-sectional study was conducted. The study protocol was approved by the Internal Review Board's Ethical Committee at Universidade Federal do Ceará (UFC), Hospital Universitário Walter Cantidio (Protocol 294/2009). All individuals provided written informed consent.

### Subjects screening and enrollment

Patients of both genders (mean age of 45.7 years old) were chosen from the outpatient service. Clinical evaluation was performed and a biochemical blood test for lactose tolerance was conducted. Blood samples were also collected for genotyping. The inclusion criteria were individuals of both genders attending the service who claimed or were shown not to be lactose intolerant but who lacked a definitive diagnosis. The exclusion criteria were individuals with other chronic clinical conditions, such as pancreatic insufficiency or lymphoma; individuals with recent use of illicit drugs, antibiotics, antiarrhythmics, hormones, corticosteroids, chemotherapeutics or other psychoactive drugs; and individuals who did not have all samples collected or who chose not to participate. Ultimately, a total of 119 subjects were enrolled in the study.

### Case definition and clinical data

The case definition was stated as patients who had self-diagnosed themselves as lactose intolerant, whereas the controls had not. Prior to performance of any laboratory tests, all patients were evaluated by a physician who checked for symptoms that define the lactose-intolerant phenotype, including borborygmus, abdominal pain, bloating, flatulence and diarrhea after ingestion of dairy products.

### Biochemical blood test

The lactose tolerance test was based on lactose ingestion. This is a widespread test used in diverse settings, including the hospital where the patients in this study were attended. After an 8-hour fast, blood samples were collected for baseline glucose determination. A 20% lactose solution was then administered orally at a dose of 2 g/kg of body weight (maximum dose of 50 g). Blood samples were again collected after 30, 60, 90 and 120 min.

The usual interpretation is as follows: an increase of less than 20 mg/dL in comparison to fasting glycemia is classified as abnormal (lactose malabsorber), whereas an increase above 25 mg/dL is considered normal and an increase between 20 mg/dL and 25 mg/dL is considered uncertain 13.

### Genotyping

For each subject, 2 mL of blood was collected for DNA sequencing and molecular analysis. Genomic DNA was extracted from blood samples following the instructions for the QIAamp DNA Blood Mini Kit (Qiagen, Valencia, CA). Polymorphism analysis was performed predominantly using restriction fragment length polymorphism/polymerase chain reaction (RFLP-PCR). DNA sequencing was performed for 20% of the samples to confirm the RFLP-PCR results.

The RFLP-PCR analysis (C>T_-13910_ SNP – rs: 4988235 and G>A-_22018_ SNP – rs: 182549) was based on the literature, with certain modifications 14,15, using the primers sense-5′-GAGTGTAGTTGTTAGACGGAG-3′ and antisense-5′-ATCAAACATTATACAAATGCAAC-3' (for C>T_-13910_ SNP) or sense-5'-AACAGGCACGTGGAGGAGTT-3' and antisense-5'-TTGAGTAGCTGGGACCACAA-3' (for G>A_-22018_ SNP) under the following PCR conditions: 95°C for 5 min, followed by 40 cycles of 95°C for 30 s, 54°C (for C>T_-13910_ SNP) or 65°C (for G>A_-22018_ SNP) for 30 s and 72°C for 1 min. The PCR products were then digested with *CviIJ* 14 or *HhaI* 15 and analyzed using 5% agarose gel electrophoresis. Digestion with *CviIJ* revealed fragments of 122 bp, 47 bp and 34 bp in the case of the C allele and fragments of 122 bp, 47 bp and 41 bp in the case of the T allele. Meanwhile, digestion with *HhaI* revealed fragments of 264 bp and 170 bp in the case of the G allele and an undigested fragment of 434 bp in the case of the A allele (**Supplemental ****Figures 1A**** and ****2A**).

**Figure 1:**
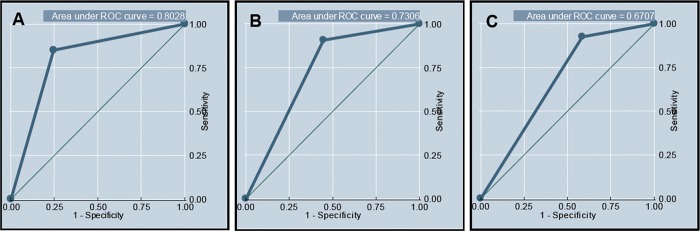
Receiver operating characteristic curve and corresponding area under the curve for the diagnosis of lactose intolerance using different cutoffs for a positive biochemical blood test: **(A)** delta <15 mg/dL, **(B)** delta <20 mg/dL and **(C)** delta <25 mg/dL, considering the lactose-intolerant phenotype.

**Figure 2:**
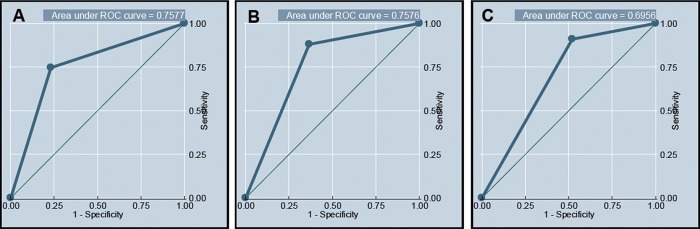
Receiver operating characteristic curve and corresponding area under the curve for the diagnosis of lactose intolerance using different cutoffs for a positive biochemical blood test: **(A)** delta <15 mg/dL, **(B)** delta <20 mg/dL and **(C)** delta <25 mg/dL, considering the single nucleotide polymorphism C>T_-13910_.

For DNA sequencing, the PCR products were purified using a QIAquick PCR Purification Kit (Qiagen, Valencia, CA) and were amplified with one of each pair of primers previously used (sense or antisense) using BigDye Terminator Sequencing Buffer with appropriate labeled ddNTPs (Applied Biosystems, Weiterstadt, Germany) under the following conditions: 96°C for 1 min, followed by 40 cycles of 96°C for 30 s, 54°C (for C>T_-13910_ polymorphism) or 65°C (for G>A_-22018_ polymorphism) for 30 s and 60°C for 4 min. After the reaction, isopropanol and 60% ethanol washes were performed and formamide was added, after which the samples were assessed using an ABI Prism 3100-Avant sequencer (Applied Biosystems, Weiterstadt, Germany) and Sequence Scanner v1.0 software (**Supplemental </!emph>**Figures 1B**** and ****2B**).**

### Statistical analysis

The data were double entered by two different persons and validated by a third person using Access and Excel 2007 software (Microsoft Inc., Redmond, WA). STATA version 8.0 software (Stata Corp LP, College Station, TX) was utilized for statistical analysis. Associations between conditions were verified by Pearson's chi-square or Fisher's exact test. Receiver operating characteristic (ROC) curves were utilized to determine which cutoff (15, 20, or 25 mg/dL) would lead to better values for sensitivity and specificity for a diagnosis of LI.

## RESULTS

### Characterization of the subjects enrolled in the study protocol

All sociodemographic parameters, such as the gender, age, ethnic group, educational status and geographic origin of the subjects included in the study, are shown in Table 1. Gender and age were discriminated by case and control group allocation, with no significant difference between them according to Fisher's exact test (*p*>0.05).

### Clinical aspects of the lactose-intolerant phenotype

The presence of symptoms that define the lactose-intolerant phenotype was assessed and compared between the case and the control groups. Overall, the most prevalent symptoms were flatulence (81.5%), bloating (68.5%), borborygmus (59.3%) and diarrhea (46.3%). Individually, flatulence, bloating, borborygmus, diarrhea and constipation symptoms were significantly associated with the case group according to Pearson's chi-squared test (*p*<0.05), whereas weight loss was not (Table 2), suggesting an efficient correlation with the lactose-intolerant phenotype.

### Biochemical blood testing and the lactose-intolerant phenotype

To improve the efficacy of the biochemical blood test, the lactose-intolerant phenotype described previously was tested for correlations with different cutoffs (delta <15 mg/dL, delta <20 mg/dL and delta <25 mg/dL) for the test for evaluation of lactose tolerance. Using delta <15 mg/dL as a positive result, 85.2% of the subjects with the lactose-intolerant phenotype were classified as lactose malabsorbers. For delta <20 mg/dL and delta <25 mg/dL, we found that 90.7% and 92.6% of subjects were lactose absorbers, respectively (Table 3).

The delta <15 mg/dL cutoff was significantly associated with the lactose-intolerant phenotype according to Fisher's exact test, which showed an odds ratio (OR) of 17.61 (95% CI: 6.88–45.06). This result provided the best cutoff in terms of sensitivity (85.2%) and specificity (75.4%) values, whereas delta <20 mg/dL yielded respective values of 90.7% and 55.4% and delta <25 mg/dL yielded respective values of 92.6% and 41.5%. For the ROC curves, delta <15 mg/dL provided an area under the curve of 80.3%, with 85.2% sensitivity and 75.8% specificity (Figure 1A), whereas delta <20 mg/dL and delta <25 mg/dL provided areas of 73.1% and 67.1%, respectively (Figures 1B and 1C).

### Genetic evaluation of the SNPs C>T_-13910_ AND G>A_-22018_

The frequency of the C>T_-13910_ polymorphism among subjects with the lactose-tolerant phenotype was 60% (39/65), with 35 CT_-13910_ genotypes and 4 TT_-13910_ genotypes. These genotypes were also observed in 24% (13/54) of the subjects with the lactose-intolerant phenotype, presenting 12 CT_-13910_ genotypes and 1 TT_-13910_ genotype. In contrast, the CC_-13910_ genotype was found in 76% (41/54) of the subjects with the lactose-intolerant phenotype, but only in 40% (26/65) of the lactose-tolerant phenotype group (Table 4). There was a significant correlation between the CC_-13910_ genotype and the lactose-intolerant phenotype as well as between the CT_-13910_ and TT_-13910_ genotypes and the lactose-tolerant phenotype according to Fisher's exact test (*p*<0.001). We found an OR of 4.731 (95% CI: 2.13–10.50) for subjects of both genders for the lactose-intolerant phenotype if they presented with the CC_-13910_ genotype.

The frequency of the G>A_-22018_ SNP was 61.5% (40/65) in the lactose-tolerant phenotype group, with 36 subjects presenting with GA_-22018_ genotypes and 4 presenting with AA_-22018_ genotypes. These genotypes were also observed in 33.3% (18/54) of subjects from the lactose-intolerant phenotype group, who presented with 17 GA_-22018_ genotypes and 1 AA_-22018_ genotype. In contrast, the GG_-22018_ genotype was found in 66.7% (36/54) of subjects with the lactose-intolerant phenotype, but not in 38.5% (25/65) of the lactose-tolerant phenotype group (Table 4). There was a significant correlation between the GG_-22018_ genotype and the lactose-intolerant phenotype as well as between the GA_-22018_ and AA_-22018_ genotypes and the lactose-tolerant phenotype according to Fisher's exact test (*p*=0.005). We found an OR of 3.20 (95% CI: 1.50–6.81) for subjects of both genders for the lactose-intolerant phenotype if they presented with the GG_-22018_ genotype.

There was no difference between observed and expected allele frequencies for the C>T_-13910_ SNP (*p*=0.911 and *p*=0.081 for the case and control populations, respectively) and G>A_-22018_ SNP (*p*=0.528 and *p*=0.056 for the case and control populations, respectively), which is consistent with Hardy-Weinberg equilibrium.

### Genetic polymorphisms and biochemical blood test correlations

Based on these results, we chose the SNP CT_-13910_ to perform a statistical analysis of the correlations between the genetic data and the three evaluated biochemical blood test cutoffs. For this analysis, we classified CT_-13910_ and TT_-13910_ as lactose-tolerant genotypes and CC_-13910_ as a lactose-intolerant genotype.

Defining delta <15 mg/dL as LM, 76.9% (40/52) of the subjects with a lactose-tolerant genotype were classified as lactose absorbers, and 74.6% (50/67) of those with a lactose-intolerant genotype were classified as lactose malabsorbers. Using delta <20 mg/dL as the cutoff, 63.5% (33/52) of the subjects with a lactose-tolerant genotype were classified as lactose absorbers and 88.1% (59/67) of those with a lactose-intolerant genotype were classified as lactose malabsorbers. Finally, for the cutoff of delta <25 mg/dL, 48.1% (25/52) of the subjects with a lactose-tolerant genotype were classified as lactose absorbers and 91.0% (61/67) of those with a lactose-intolerant genotype were classified as lactose malabsorbers. We observed a significant association with all cutoffs according to Fisher's exact test (Table 5).

According to the area under the ROC curve (75.7%), the cutoff that presented better values for sensitivity and specificity was delta <15 mg/dL. Using this cutoff, an individual could be classified as lactose intolerant with sensitivity and specificity of 74.6% and 76.9%, respectively (Figure 2A). In addition, for the cutoff of delta <20 mg/dL, sensitivity and specificity were 88.1% and 63.4%, respectively and for delta <25 mg/dL, sensitivity and specificity were 91% and 48.1, respectively (Figures 2B and 2C).

The same evaluation of the G>A_-22018_ polymorphism indicated similar correlation results (Supplemental Figure 3 and Supplemental Table 1).

### Lactose intolerance management flow chart

To optimize the clinical management of LI, we propose a flow chart for its diagnosis. As a first step, patients should undergo biochemical blood tests for evaluation of LM, interpreting delta <15 mg/dL as positive and delta >15 mg/dL as negative. If they have self-diagnosed symptoms of LI and have a positive biochemical blood test, they can be classified as lactose intolerant. In contrast, if they do not present with symptoms, they could be classified as lactose malabsorbers.

**Figure 3:**
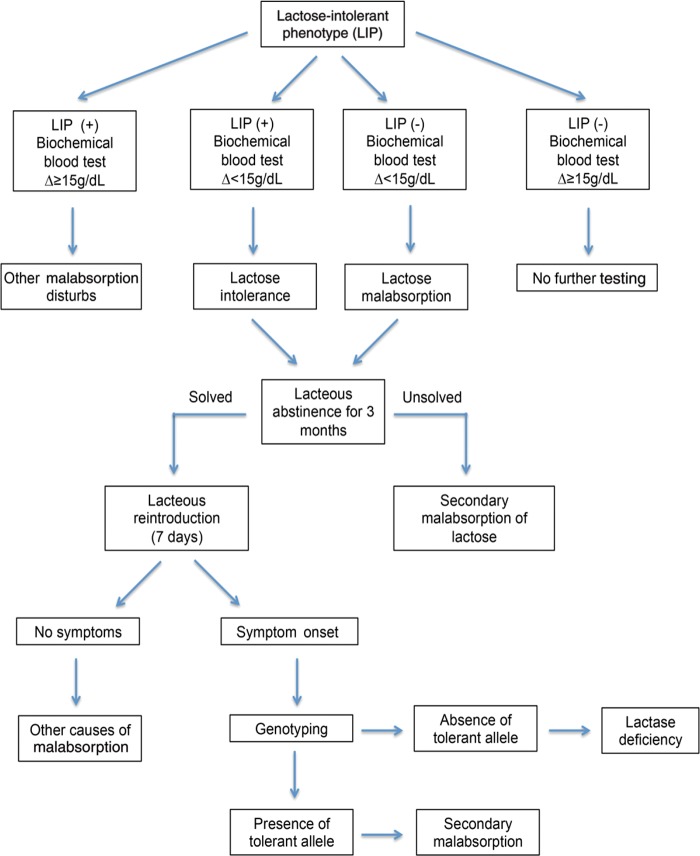
Flow chart suggesting a clinical protocol for the diagnosis of lactose intolerance by combining clinical symptoms, biochemical blood test results and single nucleotide polymorphism detection. See text for detailed description.

After excluding milk products from their diets for 3 months, individuals who continue to present with symptoms must be classified as lactose malabsorbers by secondary causes, whereas those who do not show symptoms after dietary exclusion should readopt their normal diet. If the symptoms do not return, other causes of malabsorption must be considered. If the symptoms return, genotyping of the subjects should be performed. The CC_-13910_ genotype indicates primary hypolactasia, whereas the presence of the T allele indicates secondary causes of malabsorption (Figure 3).

## DISCUSSION

LI is one of the most prevalent problems in gastrointestinal clinical practice 2. A misunderstanding of the terminology and a lack of knowledge about the multiple factors involved in the onset of symptomatology are two issues reported in the literature 1,4.

The use of a biochemical blood test to assess LM is useful, although not conclusive, as not all malabsorbers develop symptoms of LI. Many factors contribute to the development of symptoms in a patient who is a lactose malabsorber: the lactose dosage, diet, the microbiota and lactase activity in the mucosa. In this context, the clinical significance of LM or LI may be overestimated 1,6.

However, many non-malabsorbers diagnose themselves as lactose intolerant 1,6. According to the new National Institutes of Health definition, LI specifically refers to the development of symptoms after blinded lactose challenge in an individual with LM 16. We believe that it is necessary to standardize a specific clinical protocol for patients suspected of having LI, along with optimized tests for LM assessment. The present study proposes a change in the glucose level cutoff from <25 mg/dL to <15 mg/dL, as measured after lactose challenge, leading to greater levels of sensitivity and specificity.

There is a strong association between lactase persistence and ethnicity. Caucasians are more lactase persistent, whereas blacks are often hypolactasic 1. In a study performed in a Brazilian population, the investigators concluded that the T allele from the C>T_-13910_ polymorphism had similar frequencies among Brazilian White and Brown populations, whereas it was absent among Japanese Brazilians 17. Our study investigated a population composed of Whites (45.4%) and Browns (54.6%) from the State of Ceará, located in northeastern Brazil. These frequencies are consistent with the ethnic percentage population distribution in this state 18.

The use of genetic tests has been proposed for LI diagnosis in different populations to differentiate primary hypolactasia from secondary causes 19,20. Our results showed that a CC genotype is associated with LI in the population studied in the Brazilian Northeast. Conversely, the T allele (CT and TT genotypes) is associated with the lactose-tolerant phenotype. Such findings are important and suggestive for composing a future diagnostic test for hypolactasia. As already shown in other populations 9,10,21, our results also show that the C>T_-13910_ SNP is in significant agreement with the G>A_-22018_ SNP, relating CC to GG, GA to CT, and TT to AA genotypes.

In this study, the identification of individuals as genetically lactose intolerant (CC or GG), although classified as lactose tolerant based on biochemical blood tests and reported symptoms, could be explained by a slow decline in the tissue concentration of lactase, with hypolactasia developing later in life. More studies should be conducted to determine the factors involved in the variation of lactase expression throughout life. Although there is a clear association between the SNPs (C>T_-13910_ and G>A_-22018_) and lactose tolerance, the complete mechanism of lactase expression still needs to be elucidated. The interaction of transcription factors is the focus of new studies that may clarify when subjects with hypolactasic genotypes will develop LI symptoms 22. In contrast, the identification of individuals who were genetically lactose tolerant (presence of the T_-13910_ or A_-22018_ allele) but who presented with symptoms related to lactose ingestion and with biochemical blood test outcomes classifying them as malabsorbers may suggest a secondary cause of hypolactasia (such as celiac disease, gastroenteritis, parasites, or Crohn's disease, among others) related to lesions of the intestinal mucosa 10. In our study, we generally observed that such individuals were older (up to 35 years) (data not shown).

Despite the existence of a few studies that investigated these SNPs in Brazil 10,11,12, it is well known that large continental-dimension countries, such as Brazil, might show different SNP prevalences. Several recent studies from India, Israel and Colombia have also found different associations between SNPs and chemical test results among different ethnic groups within the same region 21,. Moreover, a study on African populations highlighted the limitations of the C>T_-13910_ polymorphism among people with non-European ancestry 26. In this context, a Brazilian study indicated that G>A_-22018_, but not C>T_-13910_, is the SNP that must be employed for the analysis of LI in Brazilians of Japanese descent 27. These observations highlight the need for more studies in the Northeast of Brazil.

Many studies have attempted to assess SNPs' associations with breath test and lactose tolerance test results 25,. The present study is the first study to evaluate correlations between different cutoffs for glucose levels in biochemical blood tests for lactose tolerance and genotyping of the SNPs C>T_-13910_ and G>A_-22018_ upstream of the lactase gene. After ROC curve analysis, the most applicable cutoff for LM diagnosis was delta <15 mg/dL. As the cutoff is increased (to <20 mg/dL or <25 mg/dL), the sensitivity value of the test becomes higher, whereas the specificity becomes lower. It is important to note that the biochemical blood test does not represent the gold standard for diagnosis of LM, as the intestinal microbiota, the lactose dosage, individual metabolic differences and diet might influence the results; the measurement of lactase activity through intestinal biopsies is the ideal approach, although invasive 6.

Disagreements between biochemical and genotypic tests 21 show that it is important to evaluate clinical aspects and to consider factors that cause LI other than the genetic basis. In fact, certain patients may not develop LI, despite their genetic basis; these individuals may develop symptoms later in life. We believe that these considerations are extremely important when assessing patients with gastrointestinal symptoms similar to LI.

Other studies have proposed a flow chart, but biochemical blood testing following lactose ingestion and glucose level measurement are rarely evaluated 6,31. Here, we present a diagnostic algorithm for LI, considering biochemical blood test results, clinical symptoms, and genotyping of the SNPs C>T_-13910_ and G>A_-22018_. This protocol will undoubtedly be very useful in settings where a breath test is not performed routinely.

This study has certain limitations, such as the lack of a comparison between the biochemical blood test and a breath test and the small sample number; overcoming these limitations would provide a more precise conclusion about the usefulness of current LM tests. This is therefore a pilot study that must be performed in other settings. Furthermore, investigation of other factors that modulate LI symptom onset in a cohort setting must be performed in future studies.

This study corroborates the association of the SNPs C>T_-13910_ and G>A_-22018_ with lactose tolerance in a population located in northeastern Brazil (OR=4.73 and OR=3.2, respectively). The data from this study suggest changing the delta cutoff for the biochemical blood test for LI from <25 mg/dL to <15 mg/dL based on the ROC curve using the lactose-intolerant phenotype and genetic polymorphism.

## AUTHORS CONTRIBUTION

Ponte PR conducted the clinical evaluations and biochemical testing. Medeiros PH, Cid DA, Prata MM and Havt A performed the genotyping experiments. Caetano JA, Soares AM and Lima AA analyzed the data. Medeiros PH and Lima A wrote the paper. Havt A, Guerrant RL and Mychalekyi J provided critical reading with helpful suggestions. Lima AA designed and provided funding for the study. These authors contributed equally to this work.

## Figures and Tables

**Table 1:** Sociodemographic parameters in the study population from northeastern Brazil.

Sociodemographic parameter	Cases (%) n=54	Controls (%) n=65	n (%) n=119
**Gender**			
Male	(24 (44.5)	(30 (46.2)	(54 (45.4)
Female	(30 (55.5)	(35 (53.8)	(65 (54.6)
**Age (years)**			
<15	(0 (0)	(2 (3.1)	(2 (1.7)
15–24	(4 (7.4)	(5 (7.7)	(9 (7.5)
25–34	(10 (18.5)	(12 (18.4)	(22 (18.5)
35–44	(7 (13.0)	(12 (18.4)	(19 (16.0)
45–54	(16 (29.6)	(16 (24.6)	(32 (26.9)
55–64	(10 (18.5)	(11 (17.0)	(21 (17.6)
65 or more	(7 (13.0)	(7 (10.8)	(14 (11.8)
Ethnic group*			
Browns					(65 (54.6)
Whites					(54 (45.4)
Blacks					(0 (0)
**Educational status**			
Elementary school					(4 (3.3)
Middle school					(2 (1.7)
High school					(45 (37.8)
Undergraduate school					(51 (42.9)
Graduate school					(17 (14.3)
**Geographic origin**			
Fortaleza (capital of Ceará State)					(109 (91.6)
Ceará Countryside					(10 (8.4)
Total					(119 (100)

**<?ENTCHAR ast?>:** Based on ethnic self-categorization as a White, Brown or Black Brazilian 13.

**Table 2:** Distribution of clinical symptoms among the case and control groups in the study population from northeastern Brazil.

Symptom	Cases n (%)	Controls n (%)	*p*-value*
**Flatulence**			
No	(10 (18.5)	(35 (53.8)	0.0001
Yes	(44 (81.5)	(30 (46.2)	
Total	(54 (100)	(65 (100)	
**Bloating**			
No	(17 (31.5)	(34 (52.3)	0.0264
Yes	(37 (68.5)	(31 (47.7)	
Total	(54 (100)	(65 (100)	
**Borborygmus**			
No	(22 (40.7)	(42 (64.6)	
Yes	(32 (59.3)	(23 (35.4)	0.0264
Total	(54 (100)	(65 (100)	
**Diarrhea**			
No	(29 (53.7)	(49 (75.4)	
Yes	(25 (46.3)	(16 (24.6)	0.0196
Total	(54 (100)	(65 (100)	
**Constipation**			
No	(32 (59.3)	(46 (70.8)	0.0015
Yes	(22 (40.7)	(19 (29.2)	
Total	(54 (100)	(65 (100)	
**Weight loss**			
No	(41 (75.9)	(53 (81.5)	0.5028
Yes	(13 (24.1)	(12 (18.5)	
Total	(54 (100)	(65 (100)	

**<?ENTCHAR ast?>:** Fisher's exact test

**Table 3:** Association between different cutoffs for the biochemical blood test and the case and control groups.

Different cutoffs (Delta = maximum – basal glucose concentration)	Cases n (%)	Controls n (%)	*p*-value*
**Delta <15 mg/dL**			
Lactose absorber	(8 (14.8)	(49 (75.4)	<0.0001
Lactose malabsorber	(46 (85.2)	(16 (24.6)	
Total	(54 (100)	(65 (100)	
**Delta <20 mg/dL**			
Lactose absorber	(5 (9.3)	(36 (55.4)	<0.0001
Lactose malabsorber	(49 (90.7)	(29 (44.6)	
Total	(54 (100)	(65 (100)	
**Delta <25 mg/dL**			
Lactose absorber	(4 (7.4)	(27 (41.5)	<0.0001
Lactose malabsorber	(50 (92.6)	(38 (58.5)	
Total	(54 (100)	(65 (100)	

**<?ENTCHAR ast?>:** Fisher's exact test

**Table 4:** Correlation between the genotypes for the single nucleotide polymorphisms C>T_-13910_ and G>A_-22018_ and the case and control groups.

Genotype	Cases n (%)	Controls n (%)	*p*-value*
**C>T_-13910_ SNP**			
TT	(1 (1.8)	(4 (6.2)	<0.0001
CT	(12 (22.3)	(35 (53.8)	
CC	(41 (75.9)	(26 (40.0)	
Total	(54 (100)	(65 (100)	
**G>A_-22018_ SNP**			
AA	(1 (1.8)	(4 (6.1)	0.0031
GA	(17 (31.5)	(36 (55.4)	
GG	(36 (66.7)	(25 (x)	
Total	(54 (100)	(65 (100)	

SNP: Single nucleotide polymorphism

**<?ENTCHAR ast?>:** Fisher's exact test

**Table 5:** Correlation between the biochemical blood test with different cutoffs (delta <15 mg/dL, delta <20 mg/dL or delta <25 mg/dL) and the genotype for the single nucleotide polymorphism C>T_-13910_.

Cutoff (Delta = maximum – basal glucose concentrations)	C>T_-13910_ SNP		*p*-value*
		Lactose tolerant n (%)		Lactose intolerant n (%)	
**Delta <15 mg/dL**			
Lactose absorber	40 (76.9)	17 (25.4)	<0.0001
Lactose malabsorber	12 (23.1)	50 (74.6)	
**Total**	**52 (100)**	**67 (100)**	
**Delta <20 mg/dL**			
Lactose absorber	33 (63.5)	8 (11.9)	<0.0001
Lactose malabsorber	19 (36.5)	59 (88.1)	
**Total**	**52 (100)**	**67 (100)**	
**Delta <25 mg/dL**			
Lactose absorber	25 (48.1)	6 (9.0)	<0.0001
Lactose malabsorber	27 (51.9)	61 (91.0)	
**Total**	**52 (100)**	**67 (100)**	

SNP: Single nucleotide polymorphism

**<?ENTCHAR ast?>:** Fisher's exact test

**Table 1S:** Correlation between biochemical blood test with different cutoffs (Delta < 15g/d, Delta < 20g/d or Delta < 25g/d) and genotypes from SNP G>A_-22018_.

**Cutoff** (Delta = maximum – basal glucose concentrations)	**SNP G>A_-22018_**		p value*
	**Lactose Tolerant n (%)**	**Lactose Intolerant n (%)**	
**Delta < 15 mg/dL**			
Lactose tolerant	41 (70.7)	16 (26.2)	<0.0001
Lactose intolerant	17 (29.3)	45 (73.8)	
**Total**	**58 (100)**	**61 (100)**	
**Delta < 20 mg/dL**			
Lactose tolerant	33 (56.9)	8 (13.1)	<0.0001
Lactose intolerant	25 (43.1)	53 (86.9)	
**Total**	**58 (100)**	**61 (100)**	
**Delta < 25 mg/dL**			
Lactose tolerant	25 (43.1)	6 (9.8)	<0.0001
Lactose intolerant	33 (56.9)	55 (90.2)	
**Total**	**58 (100)**	**61 (100)**	

SNP: Single Nucleotide Polymorphism

**<?ENTCHAR ast?>:** Fischer's exact test
